# MMPro-HIP: multimodal progressive fusion model for elderly HIP fracture risk prediction

**DOI:** 10.3389/fmed.2026.1721906

**Published:** 2026-04-28

**Authors:** Songyuan Chen, Ziqi Liu, Yifan Cao, Wei Wang, Yanna Lu, Shujing Lou, Jie Zi, Binghui Guo, Ziqiao Yin, Yuan Yuan, Wei Tian

**Affiliations:** 1 School of Artificial Intelligence, Beijing Advanced Innovation Center for Future Blockchain and Privacy Computing, Beihang University, Beijing, China; 2 Key Laboratory of Mathematics, Informatics and Behavioral Semantics and State Key Laboratory of Complex & Critical Software Environment, Beihang University, Beijing, China; 3 School of Mathematical Sciences, Beihang University, Beijing, China; 4 Department of Geriatrics, Beijing Jishuitan Hospital, Capital Medical University, National Center for Orthopaedics, Beijing, China; 5 Longzeyuan Community Health Service Center, Beijing, China; 6 Beijing Taikang Yanyuan Rehabilitation Hospital, Beijing, China; 7 Hangzhou Internation Innovation Institute of Beihang University, Hangzhou, China

**Keywords:** data fusion, geriatric medicine, hip fractures, machine learning, progressive fusion, risk prediction

## Abstract

**Background and objectives:**

Hip fractures, often termed the “last fracture in life,” are associated with a 20–30% one-year postoperative mortality and about 50% of survivors endure permanent disability and loss of independence. Early, accurate risk prediction enables timely preventive measures and individualized care, reducing incidence. However, older adults frequently present with mobility limitations, multiple chronic comorbidities, and heterogeneous hospital resources, leading to missing clinical or imaging data. Consequently, developing hip-fracture risk models robust to incomplete data is essential to improve generalizability, feasibility, and clinical decision-making in resource-constrained settings.

**Materials and methods:**

We retrospectively analyzed 1,287 elderly clinical records from Beijing Jishuitan Hospital, Capital Medical University, including 643 patients with hip fractures and 644 controls without fractures. Baseline variables showed notable between-group differences in sex, age, fracture history, hemoglobin levels, and bone mineral density (BMD). A global machine learning model was developed using complete-case data, with performance evaluated by accuracy and the area under the receiver operating characteristic curve (AUC). To address the frequent problem of modular missing data, we further proposed a progressive fusion model (MMPro-HIP). This model integrates features from multiple modalities stepwise, allowing for robust prediction despite incomplete information.

**Results:**

The global model reached an accuracy of 84.67% and an AUC of 0.8064. Key predictors included age, sex, BMD, and cholesterol, with the section modulus within BMD emerging as an important but previously underutilized factor. The MMPro-HIP model achieved superior performance on the independent test set, with an accuracy of 90.94% and an AUC of 0.9423. By capturing cross-modal interactions, this approach outperformed the global model. Ablation experiments confirmed the contribution of demographic variables (79.06%) and BMD measures (−6.71%) to predictive accuracy.

**Conclusion:**

MMPro-HIP showed favorable predictive performance for hip fracture risk assessment in older adults with incomplete clinical data in this single-center retrospective cohort. BMD contributed the largest performance gain, while basic demographic information alone still provided useful baseline stratification. These findings suggest that progressive multimodal fusion with residual correction may be a practical strategy for structured clinical prediction under modular missingness, although external validation is still needed.

## 1 Introduction

Hip fractures, a serious skeletal disorder predominantly affecting the elderly, are associated with high disability and mortality rates, as well as substantial healthcare costs. Often termed the “last fracture in life,” hip fractures result in a 20% one-year mortality rate post-surgery, with 50% of survivors experiencing permanent disability and loss of independence (1). Statistics indicate that hip fractures account for 7.01% of all fractures and 23.79% of fractures in the elderly (2, 3). With accelerating population aging and rising osteoporosis rates, hip fractures have become a critical public health challenge, imposing heavy medical and economic burdens on individuals and society (4–6). Thus, improving hip fracture risk prediction is paramount (7).

However, missing data is a particularly critical problem in hip fracture prediction. In the era of precision medicine, the rapid advancement of artificial intelligence (AI) has increasingly directed research attention toward small-sample, high-precision medical data (8, 9). However, practical applications face significant challenges, including missing data, insufficient standardization, poor data integration, and individual variability. Among these, missing data is particularly critical. This issue, common in statistics, is exacerbated in medical contexts due to patient-specific variations that lead to misaligned or incomplete datasets, severely undermining the effectiveness of AI in precision medicine (10). Older adults frequently experience mobility limitations and multiple chronic comorbidities, which hinder the completion of certain standardized assessments and different hospitals have different medical conditions. Due to these reasons, the issue of data missingness has also emerged in the field of precision medicine for predicting the risk of hip fractures in the elderly.

Against this backdrop, Predicting and preventing hip fractures in older adults, especially under conditions of data missingness, has emerged as an urgent public health priority.

Currently, several clinical tools are widely employed to assess hip fracture risk in the elderly without missing data:

1. Fracture Risk Assessment Tool (FRAX), developed by the WHO Collaborating Center for Metabolic Bone Diseases, is the most extensively utilized tool for predicting osteoporotic fractures, including hip fractures, over a 10-year period. It integrates clinical risk factors such as age, sex, weight, height, prior fracture history, and bone mineral density (BMD) (11), and is recommended by the osteoporosis management guidelines in China (12–14).
2. QFracture (15), based on the UK General Practice Research Database, predicts 1-year to 10-year hip fracture risk without requiring BMD measurements. While validated in meta-analyses (16), its applicability to Chinese populations remains unverified.
3. Garvan Tool, focusing on sex, age, fracture history since age 50, recent falls, and weight, the Garvan Tool estimates 5- or 10-year absolute risks of hip and other osteoporotic fractures. Unlike FRAX and QFracture, it explicitly incorporates fall frequency as a predictor (17, 18). These tools enable non-imaging-based risk assessment, facilitating fracture prediction in resource-limited settings.

However, in real-world clinical scenarios, modular data missingness—where patients lack specific diagnostic modalities—poses a critical challenge (19–21). Traditional tools face a trade-off: using more features reduces sample size, while increasing sample size limits feature availability. This dilemma renders conventional prediction methods ineffective when handling incomplete datasets (22).

Artificial intelligence has shown increasing value in orthopedic surgery and musculoskeletal risk assessment, including fracture detection, postoperative prognosis, and clinical decision support. Recent reviews have highlighted the rapid expansion of AI applications in orthopedics, while also emphasizing persistent challenges related to data heterogeneity, limited transparency, and real-world deployment (23). In parallel, multimodal fusion methods have attracted growing attention in medical AI because they can integrate complementary information from heterogeneous data sources. Progressive fusion strategies have also been explored in medical image analysis, where staged integration can improve feature interaction and representation learning (24).

However, most existing studies focus either on imaging-centered tasks or on datasets with relatively complete inputs. Comparatively less attention has been paid to structured clinical risk prediction under modular missingness, where different patients undergo different examinations and therefore contribute different subsets of modalities. In hip fracture risk prediction, this problem is particularly relevant because older adults often have incomplete laboratory or imaging records in routine practice.

To overcome these limitations, we introduce a data fusion approach for hip fracture prediction and propose a progressive multimodal fusion model to address the issue of modular data missingness (25). Inspired by the stepwise diagnostic reasoning employed by clinicians, this framework conceptualizes patient data as comprising hierarchical supplementary data layers. Complete datasets are considered “enriched” with additional layers, while incomplete ones are grouped by missingness severity. For samples with complete data, the final multi-stage fused model delivers more precise predictions, whereas for those with greater missingness, earlier-stage models provide baseline assessments. This hierarchical framework balances data utilization and feature availability in hip fracture risk prediction.

**Figure 1:**
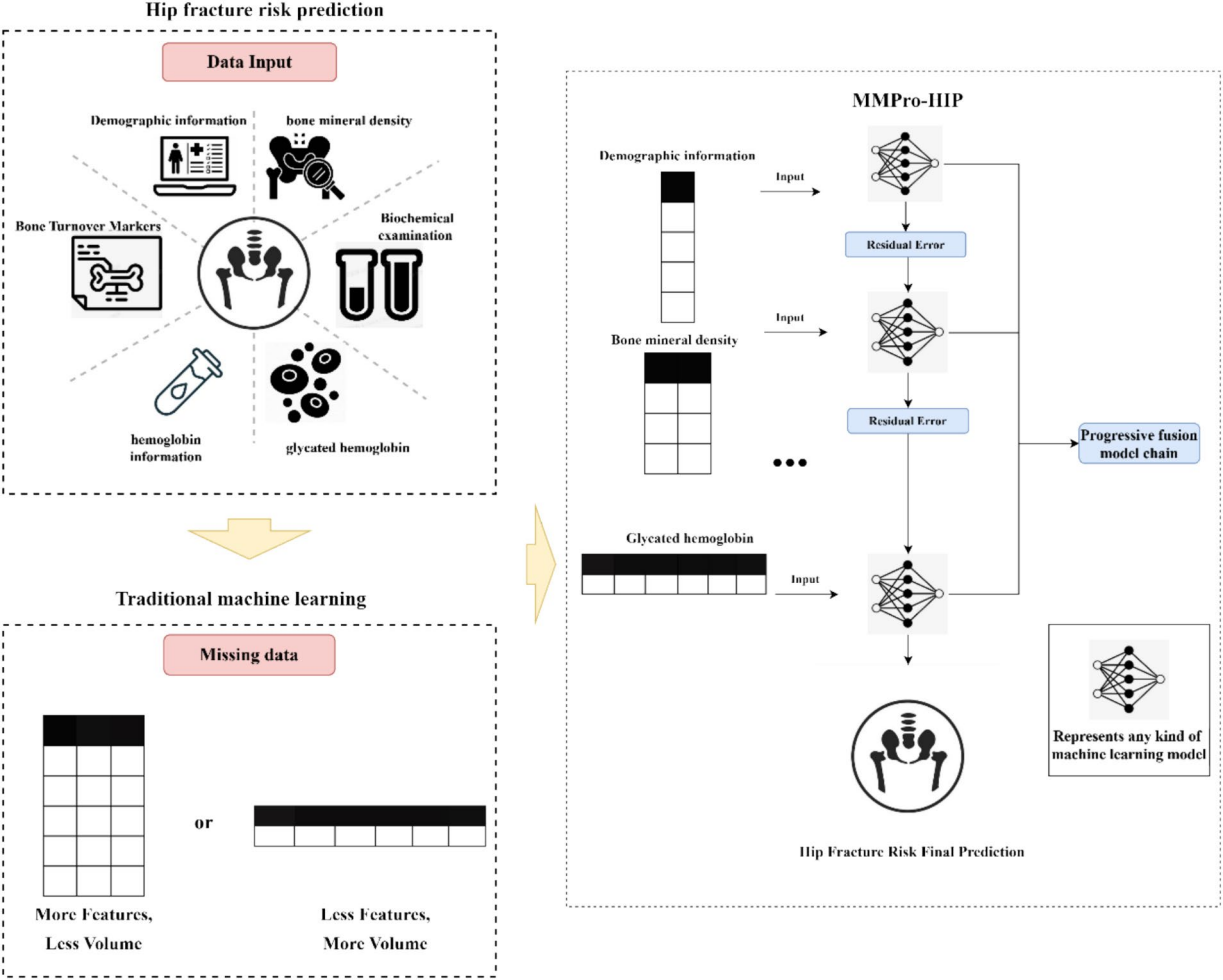
General architecture diagram.

To address modular missingness in structured clinical data, we developed MMPro-HIP, a progressive multimodal fusion framework for hip fracture risk prediction in older adults. Unlike conventional complete-case models that discard incomplete samples, or imputation-based methods that reconstruct missing values before prediction, MMPro-HIP progressively incorporates available modalities and refines predictions through residual learning. In this framework, an initial model trained on the most widely available modality provides a baseline prediction, and subsequent models iteratively learn corrective residuals from newly introduced modality groups. This design allows patients with different degrees of data completeness to be evaluated within a unified prediction framework while preserving the incremental contribution of additional clinical information (Figure 1).

## 2 Materials and methods

### 2.1 Data extraction and filtering

A total of 17,666 patient records were initially screened from Beijing Jishuitan Hospital, Capital Medical University, Capital Medical University’s database. From these, 1,287 records were extracted based on the following inclusion criteria:

1. Availability of Key Data: Records that partially or fully contained the following information:
2. Demographic Information: Gender, age, marital status, ethnicity, fracture history, smoking history (status and duration), alcohol consumption history (status and duration).
3. Bone Mineral Density (BMD) Measurements: Femoral neck BMD, femoral neck BMD T-score, total hip BMD, total hip BMD T-score, lumbar spine BMD T-score, mechanical index (MI), curvature ratio, section modulus.
4. Bone Turnover Markers: Intact parathyroid hormone (iPTH), 25-hydroxyvitamin D, total type I procollagen amino-terminal propeptide (tPINP), type I collagen carboxy-terminal telopeptide *β*-specific sequence (β-CTX), and N-terminal osteocalcin (N-MID).
5. Biochemical Examination: Serum albumin/Globulin, calcium, Alkaline phosphatase, Creatinine, total cholesterol, low-density lipoprotein cholesterol (LDL-C), high-density lipoprotein cholesterol (HDL-C), triglycerides, hemoglobin, and Glycosylated Hemoglobin (HbA1c).

All patients’ data were used anonymously, and informed consent was deemed unnecessary due to the retrospective and observational nature of the study (No. K2024-151-02).

1. Labeling of Outcome:

Samples were labeled as positive for risk prediction if the patient was diagnosed with a hip fracture within the preceding 5 years; otherwise, they were labeled as negative. This process yielded 643 positive and 644 negative samples.

### 2.2 Data processing

Each feature of the basic information was processed as follows:

1. Gender: A total of 626 male and 661 female patients were included.
2. Age: The mean age was 80.69 years.

Patients were grouped into five age categories:

- < 60 years old (*n* = 26)
- 60–70 years old (*n* = 175)
- 70–80 years old (*n* = 334)
- 80–90 years old (*n* = 631)
- > 90 years old (*n* = 121).

1. Marital status: Categorized by different marital statuses, it was divided into 4 categories: unmarried, married, divorced, and widowed.
2. Ethnicity: According to the patient’s ethnicity, it was divided into 13 groups in the order of Han, Hui, Manchu, Mongolian, Miao, Tujia, Uighur, Xibe, Kazakh, Korean, Daur, Gelao, and Zhuang.
3. Fracture history: Hip fracture history was categorized into two groups: presence or absence.
4. Smoking and Alcohol Consumption history: Both variables were recorded as binary indicators (presence or absence). For those with a history, the duration (years) of smoking and alcohol consumption is additionally documented.

Variables were preprocessed according to their data type before model construction. Binary variables, including sex, fracture history, smoking history, and alcohol consumption history, were encoded as 0/1 indicators. Multi-category variables such as marital status and ethnicity were transformed using one-hot encoding. Continuous variables, including age, laboratory measurements, and bone mineral density indicators, were retained as numeric variables. For patients with repeated measurements of the same indicator, the median value was used to represent that feature in order to reduce the influence of extreme observations. Continuous variables were standardized within the training set when required by non-tree-based baseline models.

Missing values were not globally imputed during construction of MMPro-HIP. Instead, the proposed framework handled modular missingness by allowing samples with different modality availability to enter different stages of the progressive fusion chain. For conventional baseline models, preprocessing was performed consistently using the same encoded variables, while model-specific scaling or transformation was applied only when required by the classifier implementation.

### 2.3 Overview of progressive fusion method based on residual learning

The proposed progressive fusion method is based on residual learning, in which later-stage models are designed to capture predictive information that has not been fully utilized by earlier-stage models. The core advantage of this framework is that it can accommodate different degrees of modality missingness while incorporating all samples with available non-missing information into the model chain. In practice, a baseline model first generates an initial prediction using the most widely available features, and subsequent submodels progressively refine this prediction by introducing newly available modality groups. Through this chained correction process, the framework achieves progressive optimization of prediction performance.

This process can be iteratively extended: with each additional feature group, a new sub-model is introduced to predict the residual of the prior prediction. The output of each sub-model is subsequently used to refine the preceding prediction result, thereby yielding a multi-stage, progressively optimized prediction outcome.

Let $x^{\left(1\right)},x^{\left(2\right)},\ldots ,x^{\left(T\right)}$ denote the ordered modality groups. The first-stage model $f_{1}$ is trained using the most widely available modality to produce an initial prediction:


$${\hat{y}}^{\left(1\right)}=f_{1}(x^{\left(1\right)})$$

For each subsequent stage t≥2 , the submodel $f_{t}$ is trained using the currently available modality group together with the predictive output from the previous stage, so that newly introduced information can refine the earlier prediction. This process can be written in a general recursive form as


$${\hat{y}}^{\left(t\right)}=f_{t}(x^{\left(1:t\right)},{\hat{y}}^{\left(t-1\right)})$$

Where $x^{\left(1:t\right)}$ represents the modality information available up to stage t , and ${\hat{y}}^{\left(t-1\right)}$ denotes the prediction obtained from the previous stage.

Through this chained correction process, each newly introduced modality contributes additional predictive information by refining the prediction generated at the preceding stage. During inference, the number of executed stages depends on the modalities available for a given patient. Samples with only basic information can still obtain a baseline prediction, whereas samples with more complete data can benefit from deeper progressive refinement.

A detailed mathematical derivation, training algorithm, and inference procedure are provided in the Supplementary material.

Compared with conventional single-stage fusion methods, MMPro-HIP does not require all modalities to be simultaneously present for every sample. Compared with imputation-based strategies, it avoids introducing a complete synthetic feature matrix before modeling. Instead, it preserves the original pattern of data availability and uses stage-wise residual correction to exploit the incremental value of newly available modalities. This makes the framework particularly suitable for real-world clinical settings where examination pathways differ across patients and institutions.

**Figure 2:**
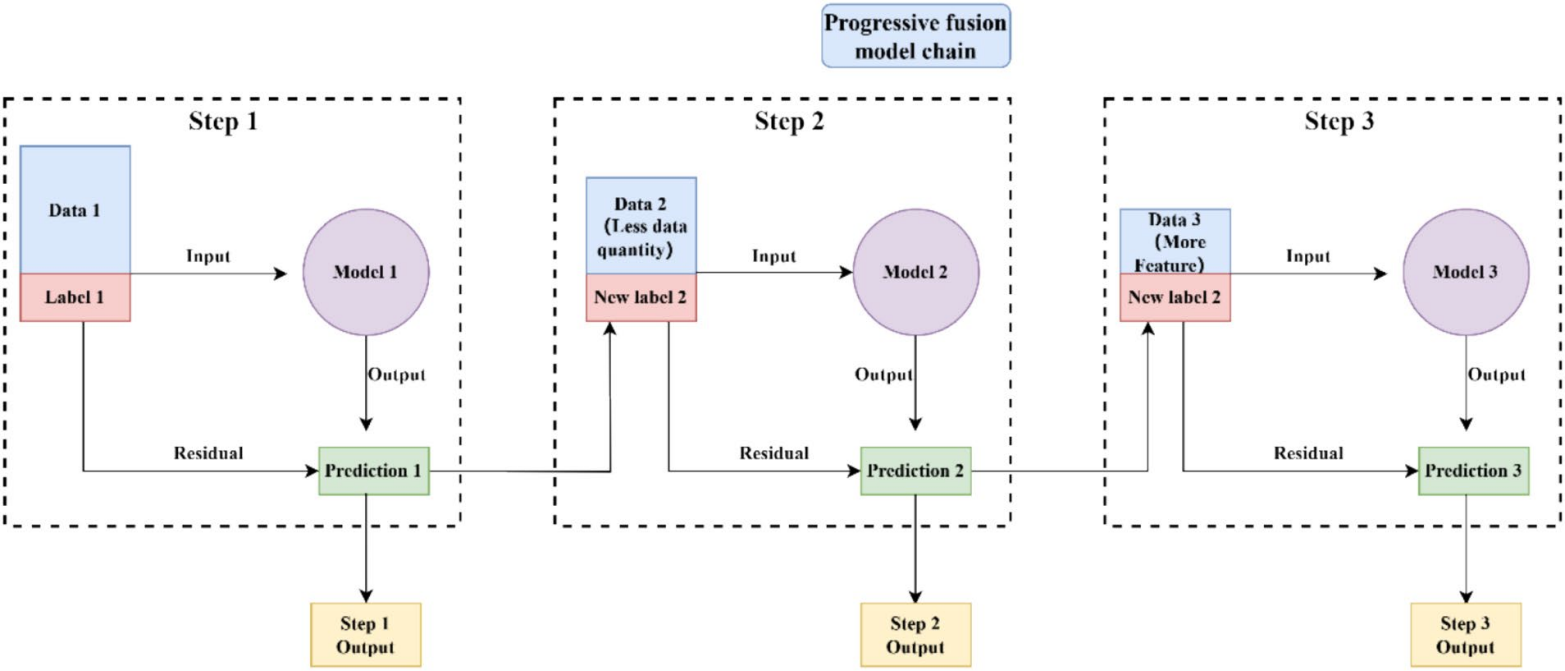
Schematic representation of the structural residual links of MMPro-HIP.

Patients with the most complete data can input all the data into the model chain to obtain more accurate predictions. Patients with less data can also obtain preliminary predictions based on the existing foundational models, thus avoiding model training issues caused by missing data structure (Figure 2).

### 2.4 Machine learning models and implementation

The second part uses the sklearn package in Python 3.10.9 to implement MMPro-HIP proposed in the paper, as well as the following baseline models: Decision Tree, Support Vector Machine (SVM), XGBoost, K-Nearest Neighbors (KNN), Naive Bayes, and Logistic Regression. For model hyperparameters not explicitly described, default values were used. The six binary classification machine learning models are as follows:

- The Decision Tree uses the DecisionTreeClassifier function from sklearn with default parameters.
- The Support Vector Machine uses the SVC function from sklearn with a polynomial non-linear kernel.
- KNN uses the KNeighborsClassifier function from sklearn with n_neighbors set to 10.
- The Naive Bayes model uses the GaussianNB function from sklearn with default parameters.
- The Logistic Regression model uses the LogisticRegression function from sklearn with default parameters.
- The XGBoost model uses the XGBClassifier function from the xgboost package, with a learning rate set to 0.1 and the number of trees set to 100.

### 2.5 Experimental setup

The proposed progressive fusion model was designed to accommodate missing feature data by progressively integrating available information.

All variables were grouped into six modality blocks according to clinical meaning and examination type: demographic information, bone mineral density (BMD), bone turnover markers, biochemical examination, hemoglobin, and HbA1c. Demographic information was placed at the first stage because it was the most complete and broadly available modality in the dataset. BMD was fixed at the second stage based on its strong association with hip fracture risk in baseline statistical analysis and prior clinical evidence. The ordering of the remaining modality groups was determined within the training set using an inner validation procedure, and the selected order was fixed before final evaluation on the independent test set.

Because the number of eligible samples decreases as more modality constraints are imposed, each stage was trained using only samples containing the current modality block together with all preceding modality blocks. To reduce class imbalance introduced by this stage-specific sample filtering process, oversampling was applied within the training set at each fusion stage. No information from the test set was used in modality ordering or stage construction.

## 3 Results

### 3.1 Basic statistical analysis

The baseline characteristics of the hip fracture and control cohorts are summarized in Table 1. Several variables showed clear descriptive differences between the two groups. Compared with the control cohort, the hip fracture cohort included a markedly higher proportion of women (79.17%) and a higher proportion of patients with previous fracture history (21.93%). Lower hemoglobin levels were also observed in the hip fracture cohort. In addition, patients in the hip fracture cohort generally showed lower values across multiple bone mineral density (BMD)-related indicators, including femoral neck BMD, femoral neck BMD T-score, total hip BMD, total hip BMD T-score, and lumbar spine BMD T-score. These descriptive findings from the full cohort are consistent in direction with the correlation analysis performed on the complete-case subset and with the subsequent model-based feature evaluation.

**Table 1:** Baseline characteristics of hip fracture and control cohorts.

Determinants	N (%)	Mean±SD, n (%)	Hip fracture cohort	Mean±SD, n (%)	Control cohort	Mean±SD, n (%)
Gender** (male)	1,287	626 (48.64%)	643	134 (20.83%)	644	492 (76.40%)
Age** (years)	1,287	80.60 ± 8.665	643	76.98 ± 8.747	644	84.38 ± 6.807
Marital status	1,287		643		644	
Ethnicity	1,287		643		644	
Fracture history (yes)	1,287	223 (17.32%)	643	141 (21.93%)	644	82 (12.73%)
Smoking history (yes)	1,287	213 (16.55%)	643	34 (5.288%)	644	179 (27.33%)
Smoking duration (years)	1,287	31.70 ± 13.36	643	35.76 ± 8.982	644	31.06 ± 15.94
Alcohol consumption history (yes)	1,287	94 (7.30%)	643	27 (4.199%)	644	67 (10.40%)
Alcohol consumption duration** (years)	1,287	31.16 ± 9.315	643	27.59 ± 6.957	644	32.63 ± 11.08
Hemoglobin (g/L)	751	117.82 ± 26.67	206	106.3 ± 30.81	425	121.4 ± 23.53
iPTH (pg/ml)	769	48.30 ± 27.14	258	49.03 ± 24.78	511	47.92 ± 28.28
25-hydroxyvitamin D (ng/ml)	769	17.58 ± 9.741	258	16.62 ± 8.891	511	18.29 ± 10.11
tPINP (ng/ml)	769	46.44 ± 29.69	258	48.81 ± 34.93	511	42.52 ± 26.45
β-CTX (ng/ml)	769	0.4362 ± 0.2720	258	0.4940 ± 0.3226	511	0.4070 ± 0.2375
N-MID (ng/ml)	769	13.86 ± 6.685	258	14.21 ± 7.749	511	13.68 ± 6.078
Serum albumin/Globulin	684	1.969 ± 4.267	176	1.954 ± 3.025	508	1.974 ± 4.643
Calcium (mmol/L)	684	2.273 ± 0.1291	176	2.283 ± 0.1174	508	2.270 ± 0.1331
Alkaline phosphatase (IU/L)	684	58.455 ± 27.19	176	63.34 ± 25.78	508	56.75 ± 27.55
Creatinine (mmol/L)	684	70.40 ± 42.60	176	67.45 ± 23.68	508	91.13 ± 47.05
Total cholesterol (mmol/L)	684	4.157 ± 1.043	176	4.468 ± 1.131	508	4.049 ± 0.9931
LDL-C (mmol/L)	684	2.219 ± 0.8179	176	2.452 ± 0.9122	508	2.138 ± 0.7664
HDL-C (mmol/L)	684	1.322 ± 0.3475	176	1.432 ± 0.3801	508	1.284 ± 0.3271
Triglycerides (mmol/L)	684	1.390 ± 1.015	176	1.327 ± 0.7557	508	1.412 ± 1.091
HbA1c (%)	665	6.520 ± 1.364	145	6.719 ± 1.477	520	6.460 ± 1.328
Femoral neck BMD**	684	0.7579 ± 0.1618	171	0.6292 ± 0.1136	513	0.8008 ± 0.1535
Femoral neck BMD T-score**	684	−1.629 ± 1.285	171	−2.551 ± 1.007	513	−1.322 ± 1.224
Total hip BMD**	684	0.8345 ± 0.1863	171	0.6675 ± 0.1300	513	0.8902 ± 0.1691
Total hip BMD T-score**	684	−1.164 ± 1.451	171	−2.391 ± 1.110	513	0.7553 ± 1.310
Lumbar spine BMD T-score**	684	0.05324 ± 2.310	171	−2.060 ± 1.623	513	0.7577 ± 2.062
MI	684	1.342 ± 0.6260	171	1.236 ± 0.5271	513	1.378 ± 0.6540
Curvature ratio	684	8.589 ± 7.680	171	9.310 ± 9.528	513	8.348 ± 6.950
Section modulus**	684	536.3 ± 234.1	171	401.3 ± 145.9	513	581.3 ± 246.8

In the “Determinants” column, variables marked with “**” were identified in the correlation analysis conducted on the complete-case subset as showing relatively strong associations with hip fracture risk. For variables with repeated measurements, the median value was used to represent each patient. Hip fracture cohort: *N* = 643; Control cohort: *N* = 644.

### 3.2 Correlation analysis and complete-case XGBoost model

Correlation analysis was further performed on the complete-case subset to evaluate the associations between individual variables and hip fracture risk. Sex (*r* = 0.5328) and alcohol consumption duration (*r* = 0.4508) showed positive correlations with hip fracture, whereas several BMD-related indicators showed moderate-to-strong negative correlations, including femoral neck BMD T-score (*r* = −0.5036), femoral neck BMD (*r* = −0.5203), total hip BMD T-score (*r* = −0.5524), lumbar spine BMD T-score (*r* = −0.5843), and total hip BMD (*r* = −0.5948). These findings indicate that lower BMD values were consistently associated with higher hip fracture risk, while sex and alcohol-related exposure also contributed meaningful predictive information. The correlation findings derived from the complete-case subset were generally consistent with the subsequent feature importance analysis and the modality ordering adopted in MMPro-HIP.

**Figure 3:**
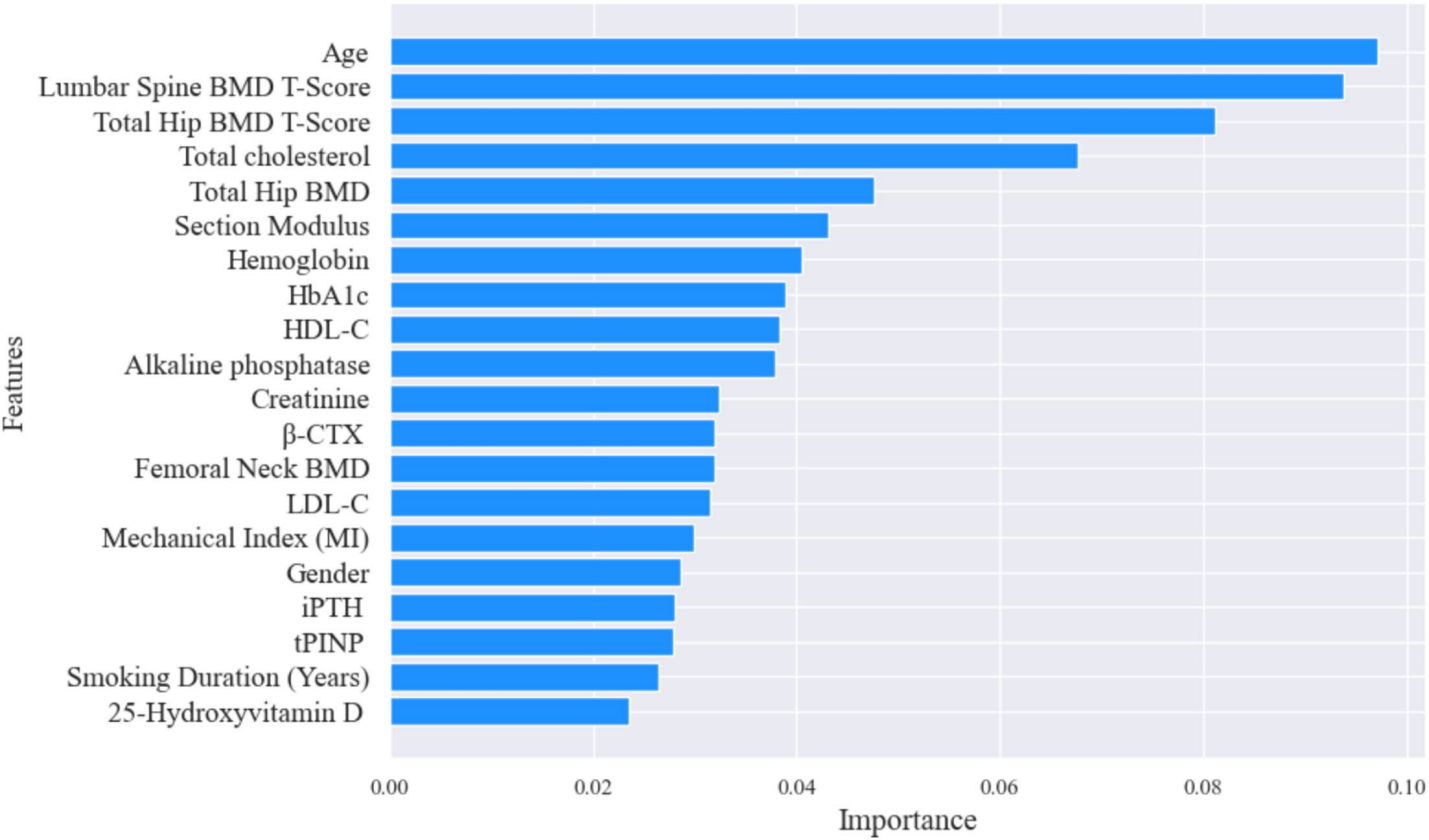
Importance analysis of features based on XGBoost model.

We trained a complete-case XGBoost model using all available features from 455 samples without missing values, achieving an accuracy of 84.67% and an AUC of 0.8064. Using the feature_importances_ function from the XGBoost package, the relative importance of features in the XGBoost model’s predictions was obtained. The results are shown in Figure 3, which displays the top 20 features in importance ranking.

Hip BMD, a key clinical indicator for hip fracture assessment (26–28), also played a critical role in predictions. Gender and age were identified as risk factors: women’s risk of hip fracture is notably higher than men’s because, over their lifetimes, women generally have lower peak bone mass and experience a rapid decline in bone density after menopause due to estrogen deficiency, which accelerates bone resorption and weakens bone microarchitecture. In addition, typical female hip geometry—narrower femoral necks and thinner cortical bone with less compensatory periosteal apposition—renders bones more susceptible to stress. Postmenopausal hormonal changes also impair muscle strength and balance, increasing fall risk, while longer female life expectancy compounds cumulative bone loss and likelihood of comorbidities (e.g., vitamin D deficiency, chronic diseases) that further undermine bone health and elevate fracture incidence (29–32), while older age amplifies risk. Further analysis reveals that certain factors in bone turnover markers and biochemical examination rank high in importance, such as those related to cholesterol. Elderly individuals often have high cholesterol levels, which has been confirmed to correlate with fracture risk. Additionally, hemoglobin and glycosylated hemoglobin impact fracture risk. Older adults at risk of hip fractures typically present with osteoporosis and impaired hematopoietic function (33). Moreover, elderly patients with poor glycemic control or those with diabetes are more susceptible to fracture risks.

### 3.3 Comparison of progressive fusion model and traditional machine learning models in predicting hip fracture risk with missing data

The training and testing data for the machine learning models are based on 1,287 medical records of hip fracture patients from Beijing Jishuitan Hospital, Capital Medical University, extracted and screened in Section Methods. The data is divided into training and testing sets in a 7:3 ratio using the train_test_split function from the sklearn.model_selection package, resulting in 900 training samples and 387 testing samples. Using the model settings machine learning models and implementation and the experimental setup from experimental setup, we train XGBoost, Decision Tree, Logistic Regression, Naive Bayes, KNN, Support Vector Machine, and MMPro-HIP. The performance differences between MMPro-HIP and the other six common machine learning models in predicting hip fracture risk are compared.

MMPro-HIP achieved optimal performance when features were integrated in the following order: basic demographic information, bone mineral density (BMD) metrics, bone turnover markers, biochemical markers, hemoglobin levels and glycosylated hemoglobin.

The risk prediction performance was evaluated based on accuracy, precision, recall, and F1-score, with results summarized in Table 2. MMPro-HIP outperformed the other six machine learning models in terms of accuracy, precision, and F1-score, while ranking second in recall. Compared to traditional machine learning models that rely solely on basic demographic information to maximize sample size, the experimental results demonstrate the superior predictive capability of MMPro-HIP for hip fracture risk assessment.

**Table 2:** Prediction efficacy for 5-year hip fracture risk.

Model	XGBoost	LR	K-nearest neighbors	Naïve Bayes	Decision tree	Support vector machine	MMPro-HIP
Accuracy	0.7804	0.7907	0.7494	0.6434	0.7933	0.8062	0.9094
Precision	0.8108	0.8148	0.8047	0.6033	0.8158	0.8019	0.9145
Recall	0.7500	0.7700	0.6800	0.9050	0.7750	0.8300	0.8669
F1-Score	0.7792	0.7918	0.7371	0.7240	0.7949	0.8157	0.8901

LR means Logistic Regression model.

**Figure 4:**
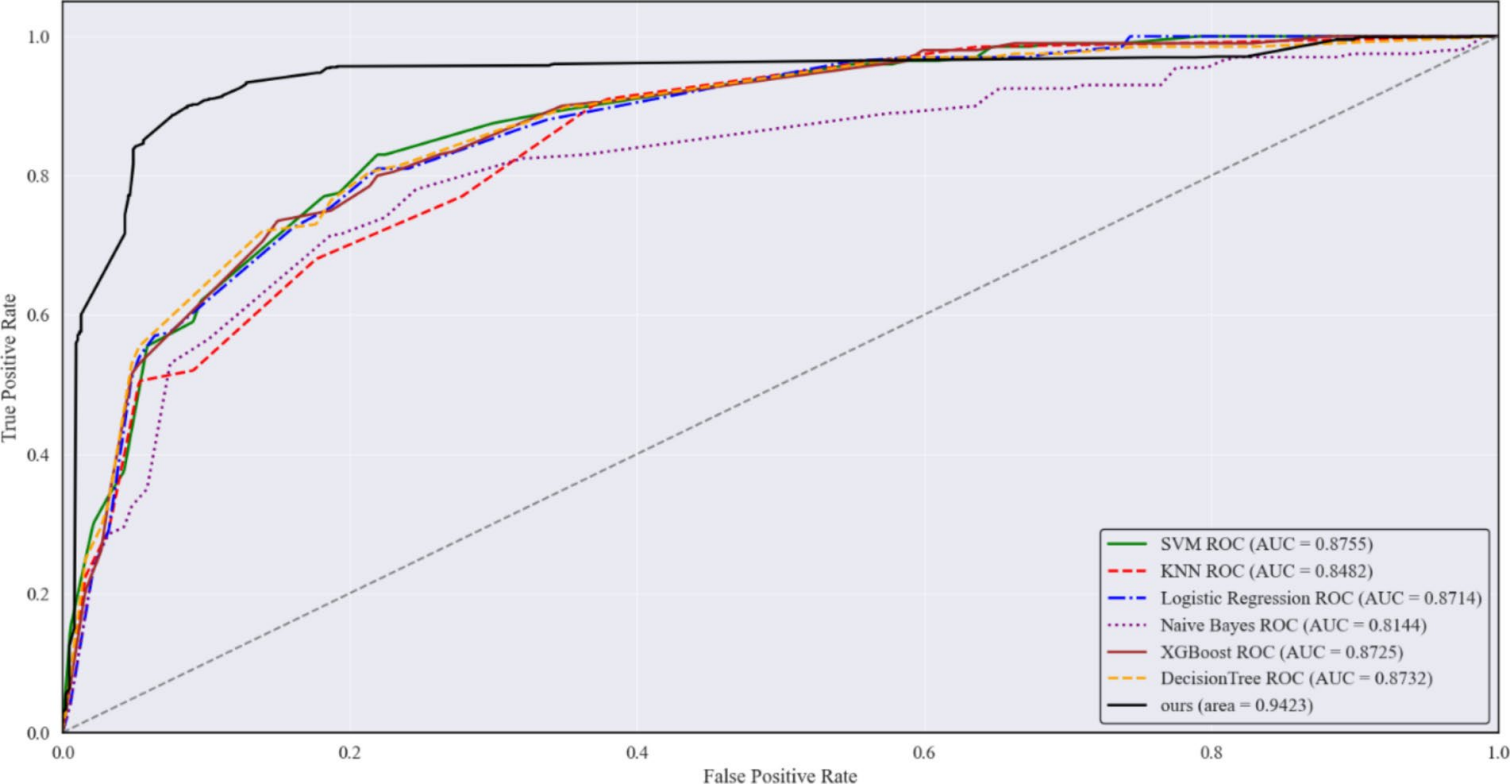
Comparison of the predicted ROC of MMPro-HIP with the remaining six models in the case of maximum data, where the solid black line is MMPro-HIP.

Further, the ROC curves for each model were plotted, as shown in Figure 4. While all six models achieved similar performance at the end of the curves, MMPro-HIP proposed in this study consistently outperformed the others across the remaining sections. From the perspective of AUC (Area Under the Curve), MMPro-HIP achieved an AUC of 0.9423(95% CI: 0.930–0.954), surpassing the other machine learning models. This further validates the effectiveness of MMPro-HIP in addressing the issue of data missingness.

These findings suggest that by integrating multiple clinical features and risk factors, the model can effectively identify elderly individuals with an elevated risk of hip fracture, thereby providing a reliable tool for early risk assessment and preventive intervention.

In this study, MMPro-HIP achieved a Brier score of 0.076, indicating a relatively small prediction error and good overall probabilistic accuracy. Since the Brier score ranges from 0 to 1 and lower values indicate better predictive performance, a value below 0.1 is generally regarded as demonstrating good prediction quality.

**Figure 5:**
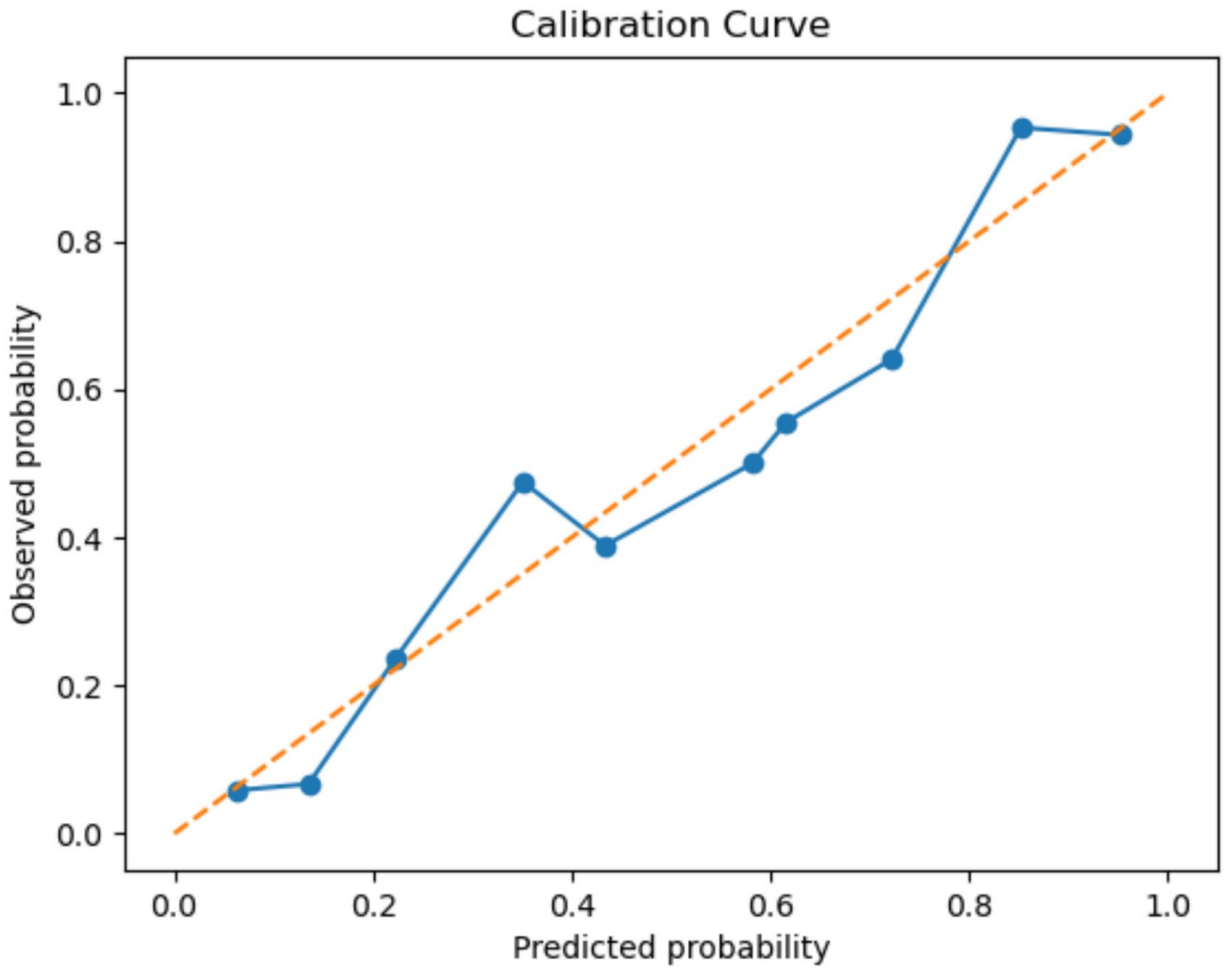
Calibration curve of MMPro-HIP.

Given that hip fractures in the elderly are associated with high morbidity, mortality, and reduced quality of life, accurate probabilistic prediction is particularly valuable for clinical decision-making and early preventive management (Figure 5).

**Figure 6:**
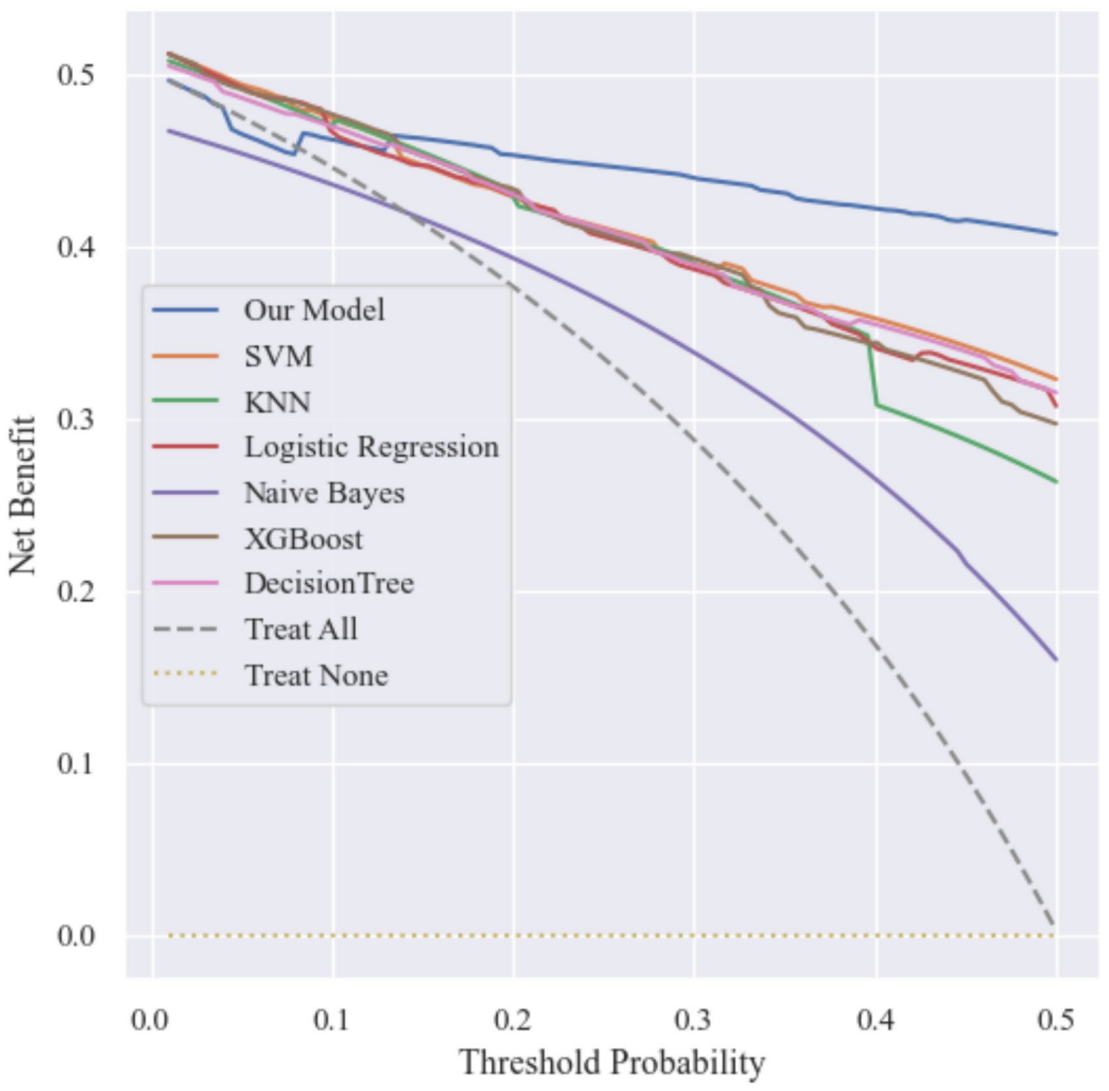
Decision curve analysis.

The calibration performance of the model was assessed using the calibration curve and the Expected Calibration Error (ECE). The model achieved an ECE of 0.025, indicating that the average deviation between predicted probabilities and observed outcomes was approximately 2.5%, which reflects good calibration performance (Figure 6).

The Decision Curve Analysis results showed that across the threshold probability range of 0 to 0.5, the proposed model consistently achieved a higher net benefit compared with both the treat-all and treat-none strategies.

Specifically, the model maintained a relatively high net benefit throughout the threshold range, indicating that using the model to guide clinical decisions could provide greater benefit than adopting extreme treatment strategies. For example, when the predicted probability exceeds a certain risk threshold, clinicians may consider implementing preventive measures such as bone mineral density screening, anti-osteoporosis treatment, fall-prevention interventions, or lifestyle modifications to reduce the likelihood of hip fracture.

These results suggest that the proposed model has promising potential for supporting clinical decision-making and improving preventive strategies for hip fractures in elderly populations.

### 3.4 Comparison of traditional machine learning models and MMPro-HIP under the most modalities

Compared to the experiments conducted in section Comparison of Progressive Fusion Model and Traditional Machine Learning Models in Predicting Hip Fracture Risk with Missing Data, this section further adjusts the training data used by the six traditional machine learning methods as baseline models. Due to data missingness, increasing the number of features necessitates a compromise on sample size. In this section, all six categories of features described in Section Data Extraction and Filtering were used for training. This setup further verifies the superiority of MMPro-HIP in predicting hip fracture risk under conditions of data missingness (Table 3).

**Table 3:** Effectiveness of hip fracture risk prediction in case of maximum number of modalities.

Model	XGBoost	LR	K-nearest neighbors	Naïve Bayes	Decision tree	Support vector machine	MMPro-HIP
Accuracy	0.8467	0.8321	0.8248	0.7532	0.8394	0.8394	0.9094

LR means Logistic Regression model.

**Figure 7:**
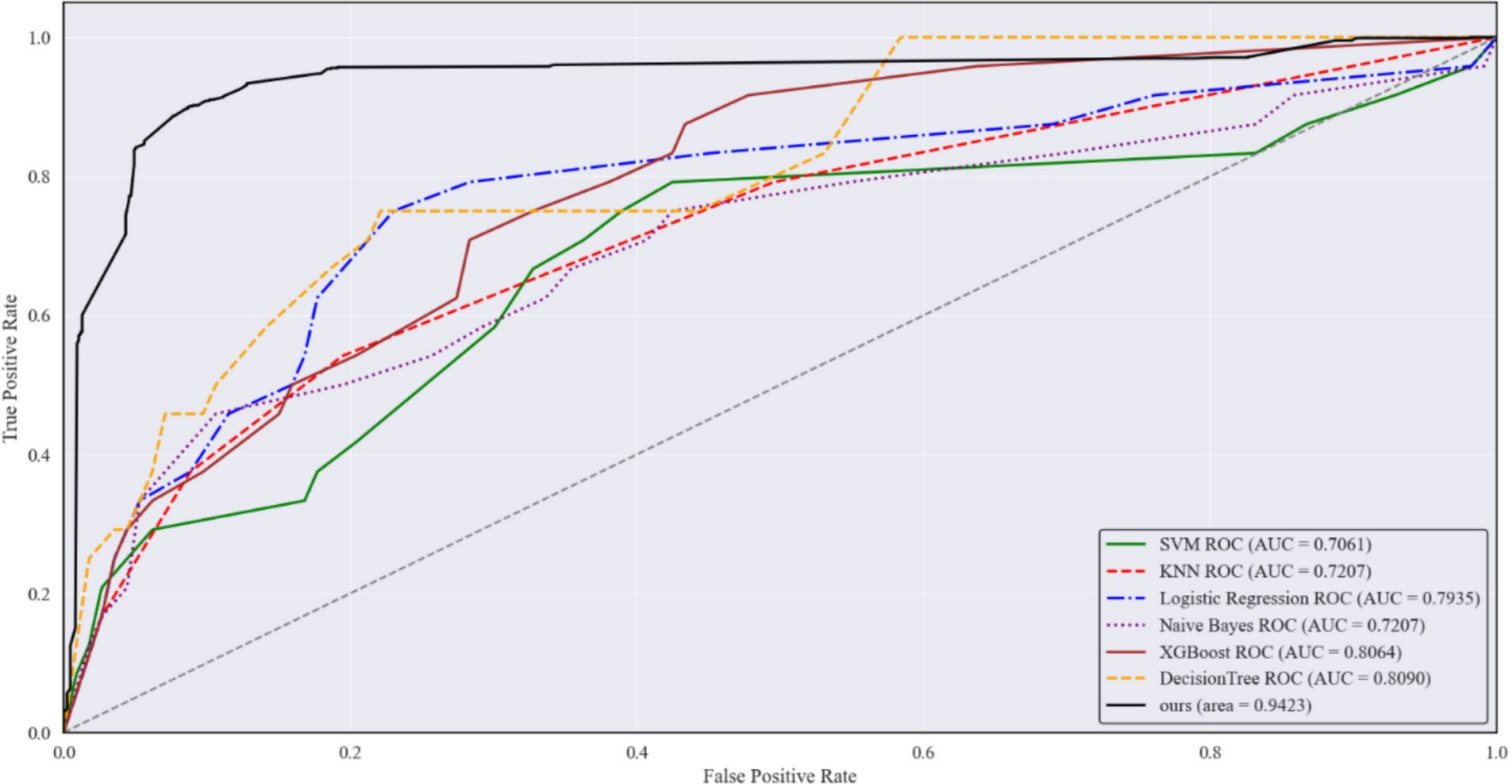
Comparison of the predicted ROC of MMPro-HIP with the remaining six models, where the solid black line is MMPro-HIP.

The ROC curves of all models are plotted in Figure 7. While all models achieved similar performance at the end of the curves, MMPro-HIP proposed in this study overall outperformed the six traditional machine learning models that rely on either maximum feature quantity or limited sample size. MMPro-HIP achieved an AUC of 0.9423, surpassing the other models.

Under conditions of modular data missingness, traditional machine learning models—whether trained with maximum sample size or maximum feature quantity—performed inferiorly compared to MMPro-HIP.

**Figure 8:**
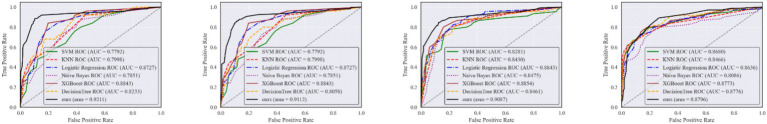
ROC curve comparison: MMPro-HIP vs. traditional machine learning under varying modality quantities. From left to right: Five modalities, four modalities, three modalities, two modalities.

As shown in Figure 8, MMPro-HIP exhibits better performance than traditional machine learning models in risk prediction ROC curves across all modality configurations, which conclusively verifies its enhanced accuracy and adaptability under varying modular complexities. The findings highlight MMPro-HIP’s enhanced capability in discriminating high-risk hip fracture cases. By dynamically integrating multi-modal clinical data through its hierarchical fusion architecture, the model robustly identifies complex interdependencies between pivotal predictors (e.g., BMD, Demographic Information, Bone markers), which improves risk stratification reliability in clinical decision-making.

### 3.5 Progressive fusion model ablation experiments

In this section, MMPro-HIP itself will be explored, and the ablation experiment will be carried out in accordance with the optimal order obtained in the previous section, to observe the impact of each part of the data on the prediction of MMPro-HIP.

In Table 4, the prediction accuracy of MMPro-HIP with varying feature quantities was analyzed by sequentially ablating each data component. The findings reveal that removing bone mineral density (BMD) metrics, bone turnover markers, or biochemical markers leads to a sharp decline in accuracy, underscoring the critical predictive role of these three categories compared to hemoglobin levels and glycosylated hemoglobin (HbA1c). This provides medical diagnostic insights for hip fracture risk prediction. Notably, even when using only basic demographic information, the model achieved an accuracy of 0.7906, further validating that basic features alone can yield relatively reliable risk predictions.

**Table 4:** Effect of MMPro-HIP hip fracture risk prediction after sequential removal of corresponding modalities, with the number of remaining feature modalities in parentheses.

Modality	−BMD (1)	−Bone turnover markers (2)	−Biochemical Examination (3)	−Hemoglobin (4)	−HbA1c (5)	All modalities (6)
Accuracy	0.7906	0.8230	0.8453	0.8804	0.8946	0.9094

The number in parentheses indicates the number of modalities whose data is used in the model. The minus sign (−) indicates the removal of that variable.

To evaluate the impact of individual feature groups, we systematically removed one category at a time while preserving the original fusion order. The resulting accuracy changes are summarized in Table 5. The experiments demonstrate that excluding BMD metrics or biochemical markers caused the most significant performance degradation, followed by bone turnover markers, while hemoglobin levels and HbA1c had comparatively minor effects. These results align with the conclusions above.

**Table 5:** Effect of progressive fusion model hip fracture risk prediction after deleting the corresponding part alone.

Modality	−BMD	−Bone turnover markers	−Biochemical examination	−Hemoglobin	−HbA1c
Accuracy	0.8423	0.8765	0.8930	0.8450	0.8946

The minus sign (−) indicates the removal of that variable.

The non-ablated full configuration of MMPro-HIP exhibits superior hip fracture risk prediction accuracy compared to its ablated variants, demonstrating the efficacy of its multi-source data integration framework. This design is particularly suited for high-precision clinical applications in tertiary hospitals. Its modular architecture further enables flexible adaptation to diverse healthcare settings: In community healthcare scenarios, cost-intensive modalities (e.g., bone mineral density and bone turnover markers) can be excluded while maintaining an accuracy range of 0.8450–0.8946 using only basic demographic data, hemoglobin, and biochemical parameters. Notably, baseline predictions relying solely on demographic information achieve 0.7906 accuracy, highlighting its adaptability. For community implementation, priority recommendations are structured as follows: Hemoglobin testing (6.44% accuracy decline upon ablation) is designated high priority due to its high clinical relevance and low cost, alongside zero-cost demographic inputs. Medium priority includes glycated hemoglobin (HbA1c), which correlates with diabetes and can be inferred from routine glucose screenings, and biochemical profiling for general health metrics. Low-priority modalities encompass BMD (6.71% accuracy decline), limited by DEXA equipment availability, and bone turnover markers (3.29% accuracy decline), constrained by specialized assay requirements. Conversely, in advanced hospital settings, full integration of all modalities achieves peak performance (accuracy: 0.8934), with BMD and bone marker integration strongly recommended for optimal outcomes.

### 3.6 Performance comparison under random forest imputation

To further investigate whether traditional machine learning models could achieve competitive performance when missing data were fully imputed, we conducted an additional experiment using a random forest–based imputation strategy. Specifically, missing values in the dataset were first completed using Random Forest imputation, after which several conventional machine learning models were trained on the completed dataset.

Table 6 summarizes the classification results of the different models after imputation. Among the traditional models, Logistic Regression and XGBoost achieved the best performance, both reaching an accuracy of 0.8527. Logistic Regression obtained a precision of 0.8454, recall of 0.8750, and F1 score of 0.8600, while XGBoost achieved a precision of 0.8557, recall of 0.8600, and F1 score of 0.8579. KNN showed relatively balanced performance with an accuracy of 0.8269 and an F1 score of 0.8378. In contrast, SVM and Naive Bayes demonstrated lower accuracy values of 0.7649 and 0.7804, respectively. Decision Tree achieved moderate performance with an accuracy of 0.8114 and an F1 score of 0.8152.

**Table 6:** Performance comparison after random forest imputation.

Model	XGBoost	LR	K-Nearest Neighbors	Naïve Bayes	Decision Tree	Support Vector Machine	MMPro-HIP
Accuracy	0.8527	0.8527	0.8269	0.7804	0.8114	0.7649	0.9094
Precision	0.8557	0.8454	0.8122	0.7805	0.8256	0.7011	0.9145
Recall	0.8600	0.8750	0.8650	0.8000	0.8050	0.9500	0.8669
F1-score	0.8579	0.8600	0.8378	0.7901	0.8152	0.8068	0.8901

Despite the use of imputation to complete missing data, the proposed MMPro-HIP model still demonstrated superior overall performance. MMPro-HIP achieved an accuracy of 0.9094, precision of 0.9145, recall of 0.8669, and F1 score of 0.8901, outperforming all traditional machine learning models across most evaluation metrics. These results indicate that even when missing values are reconstructed using imputation methods, conventional models remain limited in their ability to fully exploit heterogeneous clinical data.

**Figure 9:**
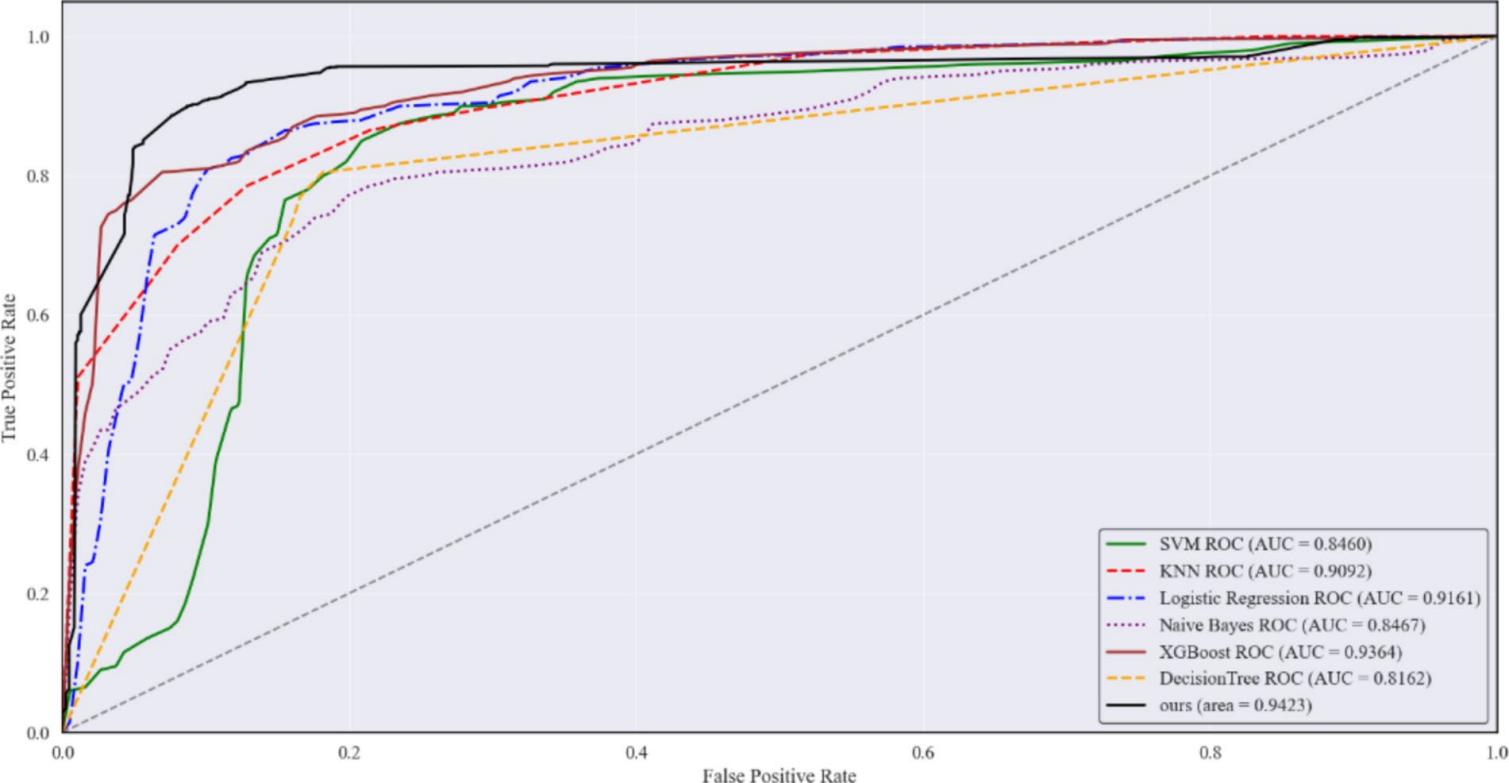
ROC curves of different models after imputation.

The receiver operating characteristic (ROC) curves for all models are presented in Figure 9. The proposed MMPro-HIP framework achieved the highest area under the curve (AUC = 0.9423, 95% CI: 0.930–0.954), slightly surpassing XGBoost (AUC = 0.9364) and Logistic Regression (AUC = 0.9161). Other models showed relatively lower discriminative ability, including KNN (AUC = 0.9092), Naive Bayes (AUC = 0.8467), SVM (AUC = 0.8460), and Decision Tree (AUC = 0.8162). Overall, the ROC analysis further confirms that the progressive multimodal fusion strategy provides stronger predictive discrimination compared with models trained on imputed datasets.

**Figure 10:**
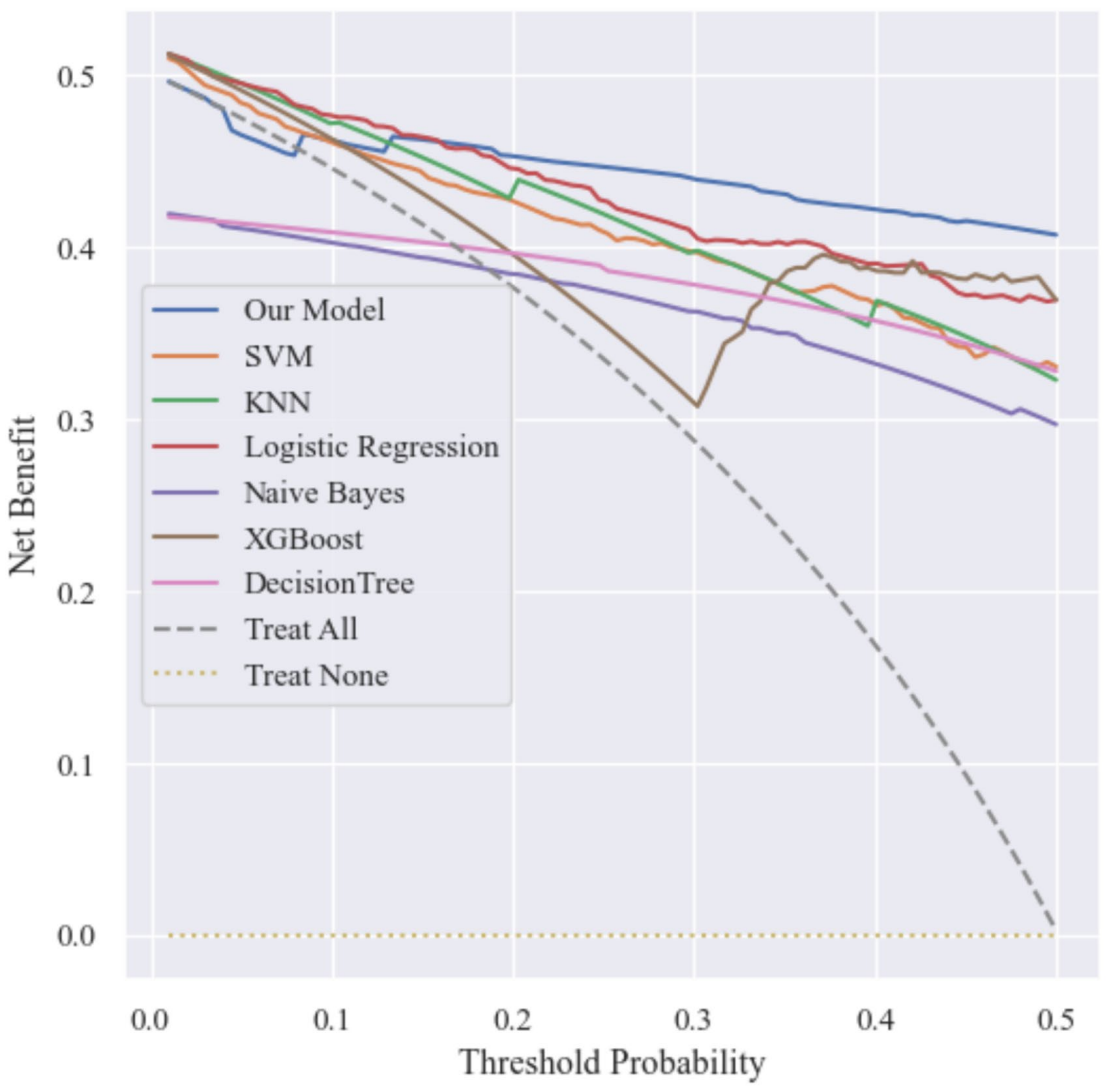
Decision curve analysis (DCA).

To evaluate the potential clinical utility of the models, decision curve analysis (DCA) was conducted, as shown in Figure 10. Across a wide range of threshold probabilities, the MMPro-HIP model consistently achieved higher net benefit compared with other machine learning models. In particular, within the clinically relevant threshold range, MMPro-HIP maintained a stable advantage over SVM, KNN, Logistic Regression, Naive Bayes, XGBoost, and Decision Tree models. This finding suggests that the proposed framework may offer greater clinical value for identifying individuals at high risk of hip fracture.

These results highlight an important limitation of relying solely on data imputation to address missingness in clinical datasets. Although random forest imputation enables traditional models to utilize a larger number of samples, the imputed values may introduce additional uncertainty or bias. In contrast, the MMPro-HIP framework directly incorporates incomplete data through its progressive multimodal fusion design, allowing the model to dynamically adapt to varying levels of data availability without relying on potentially noisy imputed values. Consequently, the proposed approach provides a more flexible and robust strategy for risk prediction in real-world clinical environments where missing data are common.

## 4 Discussion

In this study, we developed MMPro-HIP, a progressive multimodal fusion framework for predicting 5-year hip fracture risk using longitudinal clinical data. A total of 1,287 records were extracted from 17,666 patient records at Beijing Jishuitan Hospital affiliated with Capital Medical University. From an initial pool of 526 variables, features with high completeness and clinical relevance were selected and categorized into six modalities: demographic information, bone mineral density (BMD), bone turnover markers, biochemical markers, hemoglobin levels, and glycosylated hemoglobin (HbA1c).

Baseline analysis revealed several clinically meaningful differences between fracture and control groups. The fracture cohort had a substantially higher proportion of females (79.17%), consistent with the well-recognized susceptibility of women to osteoporosis-related fractures, largely due to postmenopausal estrogen deficiency and anatomical factors. Prior fracture history was also more prevalent in the fracture group, reinforcing its role as a major predictor of future fractures. In the BMD modality, fracture cases consistently exhibited lower BMD values, particularly at the femoral neck and total hip. Hemoglobin levels were also lower in fracture patients, suggesting that impaired hematopoietic function or chronic comorbidities may contribute to skeletal fragility in older adults.

To investigate the predictive value of individual features, a complete-case XGBoost model was trained using 455 samples with complete data, achieving an accuracy of 84.67% and an AUC of 0.8064. Feature correlation analysis indicated that femoral neck BMD, total hip BMD, lumbar spine BMD T-score, and section modulus were strongly negatively correlated with fracture risk, highlighting the importance of skeletal structural integrity. Feature importance analysis further showed that demographic variables and BMD metrics contributed substantially to predictive performance, while metabolic and hematological indicators such as cholesterol, hemoglobin, and HbA1c also provided complementary predictive information.

A major goal of this study was to address the challenges posed by incomplete clinical data. Traditional machine learning models often require complete datasets, resulting in a trade-off between feature diversity and sample size (34). In contrast, the proposed MMPro-HIP framework adaptively integrates available modalities through a progressive fusion strategy, enabling effective utilization of incomplete data. Compared with the best performing traditional model, MMPro-HIP achieved a classification accuracy of 90.94% and an AUC of 0.9423, demonstrating substantial improvements in predictive performance.

Ablation experiments further highlighted the importance of different data modalities. Removing BMD metrics resulted in the largest performance degradation (6.71% accuracy decline), followed by hemoglobin levels (6.44%) and bone turnover markers (3.29%). In contrast, biochemical markers and HbA1c contributed smaller but still meaningful improvements. Notably, models trained using only demographic features achieved an accuracy of 0.7906, suggesting their potential value for preliminary risk screening in resource-limited environments.

To further evaluate whether conventional approaches could achieve similar performance when missing data were fully reconstructed, an additional experiment was conducted using Random Forest-based imputation. After completing missing values, several traditional models—including Support Vector Machine, K-Nearest Neighbors, Logistic Regression, and Naive Bayes—were trained on the imputed dataset. Although imputation improved the performance of these models, the best traditional models (Logistic Regression and XGBoost) achieved accuracies of 0.8527, which remained notably lower than that of MMPro-HIP. Receiver operating characteristic analysis also showed that MMPro-HIP achieved the highest AUC (0.9423), while decision curve analysis demonstrated consistently higher net benefit across clinically relevant threshold ranges. These findings suggest that directly modeling incomplete multimodal data through progressive fusion may be more robust than relying solely on data imputation strategies.

The present study should be interpreted in the context of growing interest in AI-based orthopedic prediction and multimodal medical modeling (23). Recent evidence has shown that AI methods are increasingly being applied to orthopedic diagnosis, prognosis, and risk stratification, but their clinical implementation is often limited by data inconsistency, missingness, and limited external validation. In addition, progressive multimodal fusion has been studied in medical imaging tasks, where staged integration can improve the use of heterogeneous inputs (24). Our work differs from these studies in two important ways: first, it focuses on structured clinical risk prediction rather than image fusion; second, it explicitly addresses modular missingness by allowing prediction with different levels of modality completeness. Therefore, MMPro-HIP should be viewed as a structured-clinical extension of progressive multimodal learning, tailored to hip fracture risk assessment in real-world elderly populations.

Importantly, the proposed framework aligns well with hierarchical clinical workflows. In community healthcare settings where advanced diagnostic equipment may be limited, basic demographic information combined with inexpensive laboratory indicators such as hemoglobin may enable efficient preliminary screening. In tertiary hospitals, comprehensive clinical data—including BMD measurements obtained through Dual-energy X-ray Absorptiometry—can be incorporated to provide more precise risk predictions. This flexible configuration allows MMPro-HIP to adapt to heterogeneous healthcare environments while balancing predictive accuracy and resource efficiency.

Although MMPro-HIP showed favorable performance within our dataset, direct comparison with established tools such as FRAX, QFracture, and Garvan should be interpreted cautiously, because these tools were developed for different target populations, clinical settings, and prediction purposes (35–37). Therefore, MMPro-HIP is better considered as a complementary data-driven framework for structured multimodal clinical prediction rather than a direct replacement for existing fracture risk assessment tools.

Despite these promising findings, several limitations should be acknowledged. First, this study used a retrospective dataset collected from a single center, which may introduce potential selection bias and limit generalizability. Second, the retrospective labeling strategy may lead to possible misclassification within the control group, as some individuals classified as negative may still develop fractures outside the observation window. Third, the model has not yet been validated using external datasets, and independent multi-center studies are necessary to confirm its robustness. Finally, integrating heterogeneous clinical modalities remains technically challenging due to differences in data structure and measurement protocols across institutions.

Future work should focus on prospective validation using multi-center cohorts and explore more advanced multimodal fusion strategies to further improve model generalizability and clinical applicability.

## 5 Conclusion

In this study, we proposed MMPro-HIP, a progressive multimodal fusion framework for 5-year hip fracture risk prediction in older adults with incomplete clinical data. Using a retrospective single-center cohort, the model achieved better predictive performance than the conventional machine learning models included in this study and showed good discrimination and calibration on the internal test set. Among the evaluated modality groups, BMD-related variables contributed the largest performance gain, while basic demographic information alone still provided useful baseline stratification. These findings suggest that progressive multimodal fusion with residual correction may be a practical strategy for structured clinical prediction tasks with modular missingness. However, the present results should be interpreted within the scope of this study only. The model was developed and evaluated on a single-center retrospective dataset, and its generalizability to other populations and institutions remains to be established. Future external and prospective validation studies are needed before broader clinical application can be considered.

## Data Availability

The data analyzed in this study is subject to the following licenses/restrictions: the data used in this work was patients’ personal information and examine results. Because it involves personal privacy, it is not convenient to disclose. If necessary, you can contact the author or corresponding author to explain the specific situation and obtain the data. Requests to access these datasets should be directed to Yuan Yuan, Yuan100yuan100@163.com.
