# Supplementary material for: MMPro-HIP: multimodal progressive fusion model for elderly HIP fracture risk prediction
Source: Front Med (Lausanne). 2026 Apr 28;13:1721906. doi: 10.3389/fmed.2026.1721906 (PMC13160726; doi:10.3389/fmed.2026.1721906)
Supplement: Supplementary file 1 [file Data_Sheet_1.docx]

Supplementary Material

# Supplementary Figures and Tables

## Supplementary Figures


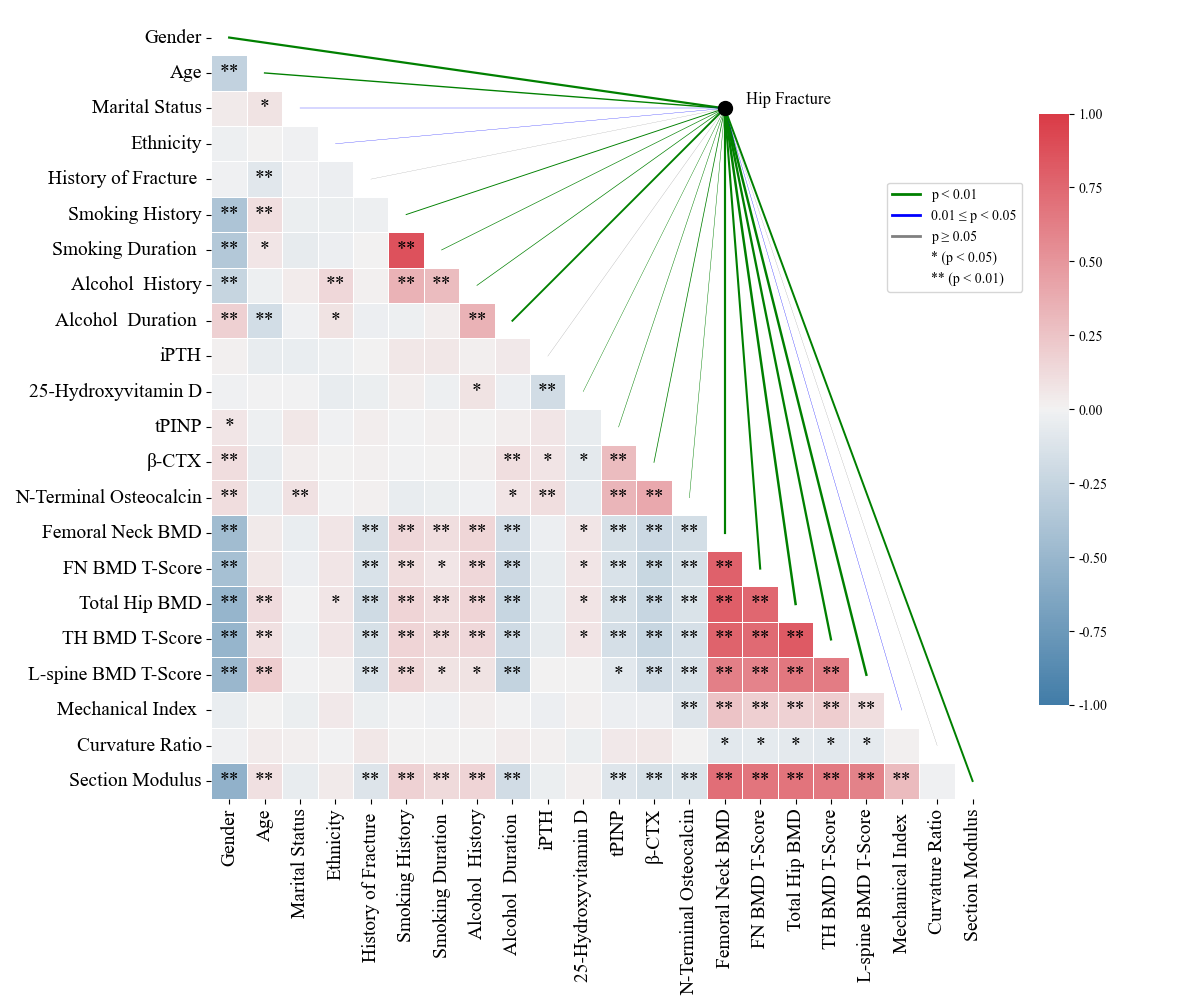


**Supplementary Figure 1.** Heatmap of correlation analysis between feature indicators and correlation analysis between feature indicators and hip fracture in elderly patients with hip fracture, the color of the squares indicates the magnitude of the correlation between features, the thickness of the line between the feature and the labeled dots in the upper right corner of the figure indicates the magnitude of the correlation between the feature and the label, the green color indicates the P-value < 0.01, the blue color indicates the P-value of 0.01 ≤ P-value < 0.05, and the gray color indicates the P-value of > 0.05

# Mathematical expression of MMPro-HIP

Suppose different samples contain different medical test data, which are divided into four categories: $\boldsymbol{\omega}_{\boldsymbol{1}}\boldsymbol{、}\boldsymbol{\omega}_{\boldsymbol{2}}\boldsymbol{、}\boldsymbol{\omega}_{\boldsymbol{3}}\boldsymbol{、}\boldsymbol{\omega}_{\boldsymbol{4}}$. To simulate the problem of data missing in small-sample medical modeling, we assume that different patients $\boldsymbol{X}_{\boldsymbol{1}}\boldsymbol{、}\boldsymbol{X}_{\boldsymbol{2}}\boldsymbol{、}\boldsymbol{X}_{\boldsymbol{3}}\boldsymbol{、}\boldsymbol{X}_{\boldsymbol{4}}$have different types of data, and $X_{1}$has four types of feature data, while the rest decrease in turn. In the first round of model training, the feature $\omega_{1}$ is used as the input, and the labels $\boldsymbol{Y}_{\boldsymbol{1}}\boldsymbol{、}\boldsymbol{Y}_{\boldsymbol{2}}\boldsymbol{、}\boldsymbol{Y}_{\boldsymbol{3}}\boldsymbol{、}\boldsymbol{Y}_{\boldsymbol{4}}$​ of the four patients are used as the output to obtain the basic model $\boldsymbol{M}_{\boldsymbol{1}}$​:

$$\boldsymbol{Y}_{\boldsymbol{i}}\boldsymbol{=}\boldsymbol{M}_{\boldsymbol{1}}\left( \boldsymbol{\omega}_{\boldsymbol{1}} \right)$$

Since $\boldsymbol{X}_{\boldsymbol{4}}$ only has the feature $\omega_{1}$, the data contained in $\boldsymbol{X}_{\boldsymbol{4}}$ will no longer participate in the training process in subsequent rounds. In the second round of model training, an incremental fusion residual learning mechanism is used. The model employs the residual learning mechanism to allow subsequent models to correct the preceding models. The specific mechanism is as follows:

In the second round of training, all three patients involved in the training have both  $\boldsymbol{\omega}_{\boldsymbol{1}}$and $\boldsymbol{\omega}_{\boldsymbol{2}}$ features. Therefore, the first set of features is input into model $\boldsymbol{M}_{\boldsymbol{1}}$ to obtain the predicted result $\boldsymbol{P}_{\boldsymbol{i}}$. The residual is then computed using $\boldsymbol{P}_{\boldsymbol{i}}$ and the true label $\boldsymbol{Y}_{\boldsymbol{i}}$ as follows, $\boldsymbol{C}$ represents the calculation of residuals:

$$\boldsymbol{E}_{\boldsymbol{1}\boldsymbol{i}}\boldsymbol{=C}\left( \boldsymbol{P}_{\boldsymbol{i}}\mathbf{，}\boldsymbol{Y}_{\boldsymbol{i}} \right)$$

$$\mathbf{C}\mathbf{=}\left\{ \begin{aligned} \mathbf{1, &}\boldsymbol{P}_{\boldsymbol{i}}\mathbf{=}\boldsymbol{Y}_{\boldsymbol{i}} \\ \boldsymbol{0}\mathbf{, &}\boldsymbol{P}_{\boldsymbol{i}}\boldsymbol{\neq}\boldsymbol{Y}_{\boldsymbol{i}} \end{aligned} \right.$$

$\boldsymbol{E}_{\boldsymbol{1}\boldsymbol{i}}$ is then used as the label for training the second model, $\boldsymbol{E}_{\boldsymbol{1}}$ represents the aggregated first-stage residual, obtained by combining the individual residuals $\boldsymbol{E}_{\boldsymbol{1}\boldsymbol{i}}$:

$$\boldsymbol{E}_{\boldsymbol{1}}\boldsymbol{=}\boldsymbol{M}_{\boldsymbol{2}}\left( \boldsymbol{\omega}_{\boldsymbol{2}} \right)$$

This results in the second model. By repeating this process, a complete model chain $\boldsymbol{M}$ is formed.

$$\boldsymbol{M=}\boldsymbol{M}_{\boldsymbol{1}}\boldsymbol{\leftarrow}\boldsymbol{M}_{\boldsymbol{2}}\boldsymbol{\leftarrow}\boldsymbol{M}_{\boldsymbol{3}}\boldsymbol{\leftarrow}\boldsymbol{M}_{\boldsymbol{4}}$$

← indicates the chained correction of the model.

During the inference phase, the proposed model employs a chained correction mechanism, wherein the total number of prediction steps is determined by the number of available feature sets. Suppose a sample contains four sets of features:  $\boldsymbol{\omega}_{\boldsymbol{1}}\boldsymbol{、}\boldsymbol{\omega}_{\boldsymbol{2}}\boldsymbol{、}\boldsymbol{\omega}_{\boldsymbol{3}}\boldsymbol{、}\boldsymbol{\omega}_{\boldsymbol{4}}$.

Initially, feature $\boldsymbol{\omega}_{\boldsymbol{1}}$ is fed into the base model $\boldsymbol{M}_{\boldsymbol{1}}$ to obtain the first-stage prediction result:

$$\boldsymbol{P}_{\boldsymbol{1}}\boldsymbol{=}\boldsymbol{M}_{\boldsymbol{1}}\left( \boldsymbol{\omega}_{\boldsymbol{1}} \right)$$

To refine this prediction using additional information, feature $\boldsymbol{\omega}_{\boldsymbol{2}}$ is input into the subsequent model $\boldsymbol{M}_{\boldsymbol{2}}$. Notably, during training, $\boldsymbol{M}_{\boldsymbol{2}}$ is supervised using the residual between $\boldsymbol{M}_{\boldsymbol{1}}$'s prediction and the true label. Accordingly, during prediction, $\boldsymbol{M}_{\boldsymbol{2}}$ outputs the estimated residual:

$$\boldsymbol{E}_{\boldsymbol{1}}\boldsymbol{=}\boldsymbol{M}_{\boldsymbol{2}}\left( \boldsymbol{\omega}_{\boldsymbol{2}} \right)$$

This residual serves as a corrective signal for the initial prediction, leading to an updated result:

$$\boldsymbol{P}_{\boldsymbol{new}\boldsymbol{1}}\mathbf{=}\left\{ \begin{aligned} \boldsymbol{P}_{\boldsymbol{1}}\mathbf{, &}\boldsymbol{E}_{\boldsymbol{1}}\mathbf{=}\boldsymbol{0} \\ \boldsymbol{-P}_{\boldsymbol{1}}\mathbf{, &}\boldsymbol{E}_{\boldsymbol{1}}\mathbf{=}\boldsymbol{1} \end{aligned} \right.$$

Here, $\boldsymbol{P}_{\boldsymbol{new}\boldsymbol{1}}$ denotes the prediction after one-stage residual correction, with $\boldsymbol{-P}_{\boldsymbol{1}}$ representing the label opposite to $\boldsymbol{P}_{\boldsymbol{1}}$. This corrected output is regarded as the result of a two-stage progressively fused model.

In addition to the stage-wise residual correction mechanism described above, two practical procedures were used to implement MMPro-HIP in the present study: determination of modality ordering and stage-wise class balancing.

To enable progressive fusion under modular missingness, MMPro-HIP was designed to incorporate all samples with available non-missing features into model training, rather than restricting the analysis to complete-case samples only. The overall training procedure consists of two key components: determination of the modality fusion order and stage-wise class balancing.

Suppose the dataset contains six feature groups, denoted by $\boldsymbol{\omega}_{\boldsymbol{1}}\boldsymbol{,}\boldsymbol{\omega}_{\boldsymbol{2}}\boldsymbol{,}\boldsymbol{\omega}_{\boldsymbol{3}}\boldsymbol{,}\boldsymbol{\omega}_{\boldsymbol{4}}\boldsymbol{,}\boldsymbol{\omega}_{\boldsymbol{5}},$and $\boldsymbol{\omega}_{\boldsymbol{6}}$. In this study, the modality with the largest sample coverage, namely basic demographic information, was used as the first stage of the model chain to train the initial submodel.

For subsequent stages, the fusion order was determined according to the number of samples retained after combining the already selected modality set with each candidate modality. Let $\boldsymbol{X}_{\boldsymbol{\omega}_{\boldsymbol{1}}\boldsymbol{,}\boldsymbol{\omega}_{\boldsymbol{i}}}$denote the subset of samples containing both $\boldsymbol{\omega}_{\boldsymbol{1}}$and $\boldsymbol{\omega}_{\boldsymbol{i}}$. The corresponding sample size is defined as

$$\boldsymbol{N}_{\boldsymbol{\omega}_{\boldsymbol{1}}}\boldsymbol{=}\max\left( \boldsymbol{sum}\left( \boldsymbol{X}_{\boldsymbol{\omega}_{\boldsymbol{1}}\boldsymbol{,}\boldsymbol{\omega}_{\boldsymbol{i}}} \right) \right)\boldsymbol{，}\boldsymbol{i}\boldsymbol{=2}\boldsymbol{，}\boldsymbol{3}\boldsymbol{，}\boldsymbol{4}\boldsymbol{，}\boldsymbol{5}\boldsymbol{，}\boldsymbol{6}$$

where $\boldsymbol{X}_{\boldsymbol{\omega}_{\boldsymbol{1}}\boldsymbol{,}\boldsymbol{\omega}_{\boldsymbol{i}}}$represents the set of samples containing both feature groups $\omega_{1}$and $\omega_{i}$, and $\boldsymbol{\sum}\boldsymbol{X}_{\boldsymbol{\omega}_{\boldsymbol{1}}\boldsymbol{,}\boldsymbol{\omega}_{\boldsymbol{i}}}$denotes the number of such samples. Thus, $N_{\omega_{1}}$is the maximum retained sample size among all candidate combinations between $\omega_{1}$and one of the remaining modality groups. The modality corresponding to this maximum value was selected as the input feature group for the second stage of progressive fusion.

The ordering of the remaining modalities was determined in the same manner. When two candidate modalities resulted in similar retained sample sizes, preliminary training performance was additionally considered. Specifically, the candidate modality yielding better overall predictive performance of the progressive fusion framework was selected for the next stage. Therefore, the final modality order was determined jointly based on sample retention, correlation analysis, and feature importance analysis.

During progressive fusion, the number of eligible samples decreases as additional modality constraints are imposed. As a result, substantial class imbalance between positive and negative samples may arise at specific training stages. To alleviate this issue, oversampling was applied to the training data at each fusion stage.

Let $\boldsymbol{N}_{\text{positive}}$and $\boldsymbol{N}_{\text{negative}}$denote the numbers of positive and negative samples, respectively. The target sample size after oversampling is defined as

$$\boldsymbol{N}_{\boldsymbol{m}}\boldsymbol{=}\max\left( \boldsymbol{N}_{\boldsymbol{positive}}\boldsymbol{，}\boldsymbol{N}_{\boldsymbol{negative}} \right)$$

where $\boldsymbol{N}_{\boldsymbol{m}}$denotes the unified class size after oversampling. The minority class was then expanded until both classes reached the same sample size $\boldsymbol{N}_{\boldsymbol{m}}$.

For categorical features, minority-class samples were first duplicated, after which selected categorical variables were randomly perturbed by assigning alternative category values within the valid feature domain, thereby generating additional synthetic samples.

For continuous features, minority-class samples were duplicated and perturbed by adding Gaussian noise. Specifically, let $\boldsymbol{X}_{\text{old}}$denote the original sample and $\boldsymbol{X}_{\text{new}}$denote the generated sample. The perturbation was defined as

$$\boldsymbol{X}_{\boldsymbol{new}}\boldsymbol{=}\boldsymbol{X}_{\boldsymbol{old}}\boldsymbol{+\epsilon,\epsilon\sim}\mathcal{N}\left( \boldsymbol{0,\tau} \right)$$

where $\boldsymbol{\epsilon}$is a random noise term sampled from a Gaussian distribution with mean 0 and variance $\tau$, and $\tau$is estimated from the observed continuous variables in the corresponding training data. In this way, additional minority-class samples were generated while preserving the overall distributional characteristics of the original data.

To avoid information leakage, this oversampling procedure was performed only within the training set at each fusion stage and was not applied to the validation or test data.

**Algorithm S1. Stage-wise training procedure of MMPro-HIP based on residual learning**

**Input:** Training dataset $\boldsymbol{D=\{(}\boldsymbol{X}_{\boldsymbol{i}}\boldsymbol{,}\boldsymbol{Y}_{\boldsymbol{i}}\boldsymbol{)}{\boldsymbol{\}}}_{\boldsymbol{i=1}}^{\boldsymbol{N}}$; ordered modality set $\boldsymbol{\Omega}\boldsymbol{=\{}\boldsymbol{\omega}_{\boldsymbol{1}}\boldsymbol{,}\boldsymbol{\omega}_{\boldsymbol{2}}\boldsymbol{,}\boldsymbol{\omega}_{\boldsymbol{3}}\boldsymbol{,}\boldsymbol{\omega}_{\boldsymbol{4}}\boldsymbol{\}}$**.**

**Output:** Progressive model chain $\boldsymbol{M=\{}\boldsymbol{M}_{\boldsymbol{1}}\boldsymbol{,}\boldsymbol{M}_{\boldsymbol{2}}\boldsymbol{,}\boldsymbol{M}_{\boldsymbol{3}}\boldsymbol{,}\boldsymbol{M}_{\boldsymbol{4}}\boldsymbol{\}}$**.**

**Procedure:**

1. Construct the first-stage training subset $D_{1}$using all samples containing modality $\omega_{1}$.
2. Train the base model $M_{1}$using modality $\omega_{1}$as input and the ground-truth label $Y$as output:

$$\boldsymbol{Y=}\boldsymbol{M}_{\boldsymbol{1}}\boldsymbol{(}\boldsymbol{\omega}_{\boldsymbol{1}}\boldsymbol{)}$$

3. For each subsequent stage $t=2,3,4$, construct the stage-specific training subset $D_{t}$using all samples containing modalities $\left\{ \boldsymbol{\omega}_{\boldsymbol{1}}\boldsymbol{,}\boldsymbol{\omega}_{\boldsymbol{2}}\boldsymbol{,\ldots,}\boldsymbol{\omega}_{\boldsymbol{t}} \right\}$**.**
4. For each sample in $D_{t}$, use the previous-stage model to obtain the prediction $P_{t-1,i}$.
5. Compute the residual label for each sample:

$$\boldsymbol{E}_{\boldsymbol{t-1,i}}\boldsymbol{=C(}\boldsymbol{P}_{\boldsymbol{t-1,i}}\boldsymbol{,}\boldsymbol{Y}_{\boldsymbol{i}}\boldsymbol{)}$$

where $\boldsymbol{C(\cdot)}$denotes the residual operation between the previous-stage prediction and the corresponding ground-truth label.

1. Merge all residual labels $\boldsymbol{E}_{\boldsymbol{t-1,i}}$into the stage-wise residual set $\boldsymbol{E}_{\boldsymbol{t-1}}$.
2. Train the current submodel $M_{t}$using the newly introduced modality $\omega_{t}$as input and $E_{t-1}$as output:

$$\boldsymbol{E}_{\boldsymbol{t-1}}\boldsymbol{=}\boldsymbol{M}_{\boldsymbol{t}}\boldsymbol{(}\boldsymbol{\omega}_{\boldsymbol{t}}\boldsymbol{)}$$

3. Repeat the above process until all modalities have been incorporated.
4. Return the complete model chain:

$$\boldsymbol{M=}\boldsymbol{M}_{\boldsymbol{1}}\boldsymbol{\leftarrow}\boldsymbol{M}_{\boldsymbol{2}}\boldsymbol{\leftarrow}\boldsymbol{M}_{\boldsymbol{3}}\boldsymbol{\leftarrow}\boldsymbol{M}_{\boldsymbol{4}}$$

**Algorithm S2. Stage-wise inference procedure of MMPro-HIP based on chained residual correction**

**Input:** A test sample $X$; available modality subset $\boldsymbol{\Omega}_{\boldsymbol{X}}\boldsymbol{=\{}\boldsymbol{\omega}_{\boldsymbol{1}}\boldsymbol{,}\boldsymbol{\omega}_{\boldsymbol{2}}\boldsymbol{,\ldots,}\boldsymbol{\omega}_{\boldsymbol{K}}\boldsymbol{\}}$, where $\boldsymbol{K\leq4}$; trained model chain $\boldsymbol{M=\{}\boldsymbol{M}_{\boldsymbol{1}}\boldsymbol{,}\boldsymbol{M}_{\boldsymbol{2}}\boldsymbol{,}\boldsymbol{M}_{\boldsymbol{3}}\boldsymbol{,}\boldsymbol{M}_{\boldsymbol{4}}\boldsymbol{\}}$**.**

**Output:** Final prediction $P_{K}$.

**Procedure:**

1. Use modality $\boldsymbol{\omega}_{\boldsymbol{1}}$as input to the base model $\boldsymbol{M}_{\boldsymbol{1}}$to obtain the first-stage prediction:

$$\boldsymbol{P}_{\boldsymbol{1}}\boldsymbol{=}\boldsymbol{M}_{\boldsymbol{1}}\boldsymbol{(}\boldsymbol{\omega}_{\boldsymbol{1}}\boldsymbol{)}$$

2. If the sample contains only one modality, output $\boldsymbol{P}_{\boldsymbol{1}}$directly.
3. For each subsequent stage $\boldsymbol{t=2,\ldots,K}$, use modality $\omega_{t}$as input to the current submodel $\boldsymbol{M}_{\boldsymbol{t}}$to obtain the residual output:

$$\boldsymbol{E}_{\boldsymbol{t-1}}\boldsymbol{=}\boldsymbol{M}_{\boldsymbol{t}}\boldsymbol{(}\boldsymbol{\omega}_{\boldsymbol{t}}\boldsymbol{)}$$

4. Correct the previous-stage prediction according to the residual output:

$$\boldsymbol{P}_{\boldsymbol{t}}\boldsymbol{=}\left\{ \begin{matrix} \boldsymbol{P}_{\boldsymbol{t-1}}\boldsymbol{,} & \boldsymbol{E}_{\boldsymbol{t-1}}\boldsymbol{=0} \\ \mathbf{opposite}\boldsymbol{(}\boldsymbol{P}_{\boldsymbol{t-1}}\boldsymbol{),} & \boldsymbol{E}_{\boldsymbol{t-1}}\boldsymbol{=1} \end{matrix} \right.$$

where $\mathrm{opposite}\boldsymbol{(}\boldsymbol{P}_{\boldsymbol{t-1}}\boldsymbol{)}$denotes the class label opposite to the previous-stage prediction.

1. Repeat the above correction process until all available modalities have been used.
2. Output the final prediction $\boldsymbol{P}_{\boldsymbol{K}}$**.**
